# Gastrointestinal Parasites in Owned Dogs in Serbia: Prevalence and Risk Factors

**DOI:** 10.3390/ani14101463

**Published:** 2024-05-14

**Authors:** Nemanja M. Jovanovic, Olga Bisenic, Katarina Nenadovic, Danica Bogunovic, Milan Rajkovic, Milan Maletic, Milorad Mirilovic, Tamara Ilic

**Affiliations:** 1 Department of Parasitology, Faculty of Veterinary Medicine, University of Belgrade, Bul. Oslobodjenja 18, 11000 Belgrade, Serbia; nmjovanovic@vet.bg.ac.rs (N.M.J.); danicab@vet.bg.ac.rs (D.B.); tamara@vet.bg.ac.rs (T.I.); 2 Faculty of Veterinary Medicine, University of Belgrade, Bul. Oslobodjenja 18, 11000 Belgrade, Serbia; olga.bisenic@gmail.com; 3 Department of Animal Hygiene, Faculty of Veterinary Medicine, University of Belgrade, Bul. Oslobodjenja 18, 11000 Belgrade, Serbia; katarinar@vet.bg.ac.rs; 4 Department of Reproduction, Fertility and Artificial Insemination, Faculty of Veterinary Medicine, University of Belgrade, Bul. Oslobodjenja 18, 11000 Belgrade, Serbia; maletic@vet.bg.ac.rs; 5 Department of Economics and Statistics, Faculty of Veterinary Medicine, University of Belgrade, Bul. Oslobodjenja 18, 11000 Belgrade, Serbia; mija@vet.bg.ac.rs

**Keywords:** dogs, helminths, protozoa, zoonoses, risk factors

## Abstract

**Simple Summary:**

This research conducted in Serbia aimed to identify intestinal parasites in dogs that could potentially infect humans. Total prevalence of intestinal endoparasites was 62.6%. Various endoparasites such as *Cystoisospora* spp., *Sarcocystis* spp., *Neospora caninum*/*Hammondia* spp., *Giardia intestinalis*, *Toxocara canis*, *Toxascaris leonina*, Ancylostomatidae, *Trichuris vulpis*, *Capillaria* spp., *Alaria alata* and Taeniidae were found. Factors like age, outdoor living, attitude and diet were linked to higher infection rates. This study emphasizes the importance of educating dog owners, conducting routine parasitological tests on their pets and regular deworming strategies.

**Abstract:**

Dogs are the most popular pets worldwide. Close contact between dogs and people increases the risk of transmission of various zoonotic parasitic infections. Given the importance of veterinary medicine in preserving the One Health concept, the aim of this research was to identify intestinal parasites that may have zoonotic potential and to evaluate risk factors (individual and environmental). The research was conducted in Serbia in 2022 and 2023 on 382 owned dogs, using qualitative methods of coprological examination with a concentration on parasitic elements. The overall prevalence of intestinal parasites was 62.6%, with the following detected: protozoa: *Cystoisospora* spp. (9.2%), *Sarcocystis* spp. (4.5%), *Neospora caninum*/*Hammondia* spp. (3.7%), *Giardia intestinalis* (11.8%); nematoda: *Toxocara canis* (11.5%), *Toxascaris leonina* (4.2%), family Ancylostomatidae (38.0%), *Trichuris vulpis* (21.5%), *Capillaria* spp. (10.5%); trematoda: *Alaria alata* (1.6%) and cestodes from the Taeniidae family (1.3%). Factors like age, size and coat length, as well as the way of living, attitude and diet were linked to a significantly higher (*p* < 0.05) prevalence of intestinal parasites. Based on the results of coprological diagnostics, this research indicates the importance of educating dog owners, conducting routine parasitological tests on their pets and regular deworming strategies.

## 1. Introduction

Among social animals, dogs are considered the most popular pets worldwide. Over the last decade, the interaction between humans and dogs has significantly increased, leading to these animals being treated as equal members of the family [1]. Such interactions may pose a risk of transmitting zoonotic pathogens. Dogs can be infected with different intestinal parasites, including protozoa (*Giardia intestinalis*, *Cystoisospora* spp., *Sarcocystis* spp., *Neospora caninum*) and helminths (roundworms, hookworms, whipworms and tapeworms) [2,3,4,5,6,7,8,9]. Clinical symptoms depend primarily of the dog’s health, the type and severity of the parasite infection and the presence of additional parasitic infections in other organ systems (e.g., cardiorespiratory, urinary). However, infections are often asymptomatic.

Various studies conducted worldwide report a high prevalence of different types of parasites in the category of owned dogs [2,7,10,11,12]. Accordingly, they may serve as reservoirs of zoonotic parasites and can contaminate soil with the infectious stages of the parasites, such as the eggs and larvae of helminths, as well as the cysts of protozoa [13,14,15,16,17]. Some dog parasites can also infect humans, causing disease. Infection can occur either directly (i.e., trophically) and/or indirectly through contaminated food and water in the environment [18,19]. The risk of infection depends on various factors, both biological and environmental, which vary based on the parasite’s life cycle and human behavior. Studies indicate that many pet owners are unaware of how dog endoparasites are transmitted and the public health risks they pose [10,11,20].

Knowing the epidemiological situation of intestinal parasites in dogs and identifying the ways they spread are key elements for effectively monitoring this threat. Bearing in mind the importance of veterinary medicine in maintaining the One Health concept, and recognizing the role of dogs in the spread of parasitic zoonoses, the aim of this study was to (i) identify gastrointestinal (GI) parasites in owned dogs using coprological diagnostics and (ii) to assess the risk factors important for the occurrence, maintenance and spread of parasitic infections.

## 2. Material and Methods

### 2.1. Study Area

**Figure 1:**
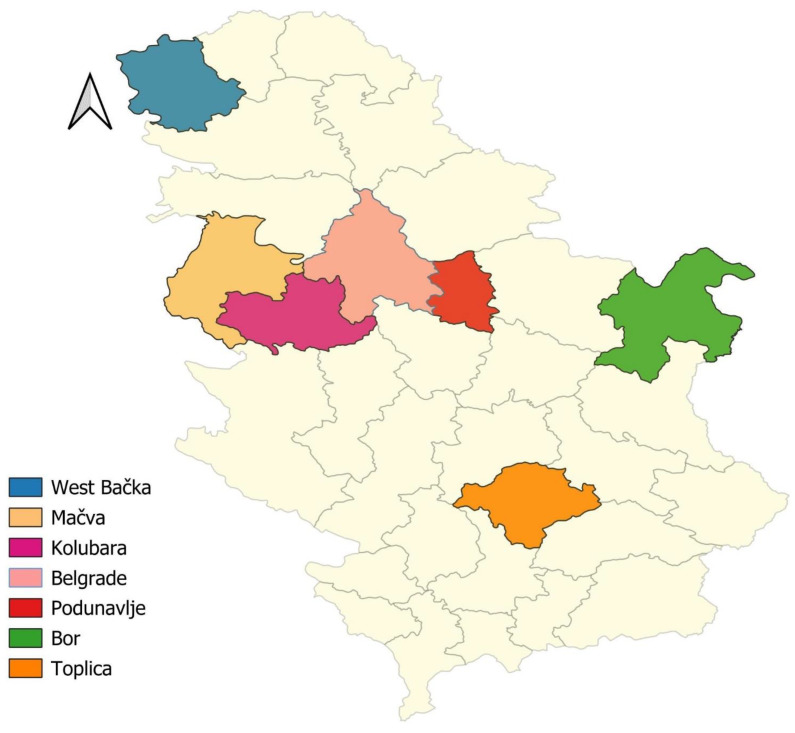
Map of Serbia with administrative districts where the survey was conducted. The map was generated by using QGIS v3.36 [21].

The survey was conducted from November 2022 to June 2023 in seven administrative districts in the Republic of Serbia: Belgrade, Podunavlje, Kolubara, Mačva, West Bačka, Toplica and Bor (Figure 1). Serbia is a landlocked country located in the Balkan peninsula and the Pannonian Plain. Serbia lies between latitudes 41° and 47° N, and longitudes 18° and 23° E. In the northern part of the country, the climate is more continental, with colder winters and warmer summers, while in the southern part, the climate tends to be more Mediterranean, with milder winters and hotter summers. The average annual rainfall ranges from around 600 to 1000 mm. The average elevation of Serbia is approximately 500 m above sea level.

### 2.2. Coproparasitological Examination

A total of 382 fecal samples were collected from owned dogs. The samples were stored at +4 °C in labeled disposable containers and transported to the Department of Parasitology at the Faculty of Veterinary Medicine, University of Belgrade, for parasitological analysis. Coproparasitological examination included the assessment of samples using both macroscopic and microscopic methods. Macroscopic examination was used to evaluate the presence of adult nematodes and proglottids of tapeworms (described in Ilić et al. [3]). For microscopic examination, samples (approximately 5–10 g per sample) were prepared using qualitative coprological diagnostic procedures—centrifugal flotation with saturated zinc sulphate solution (with a specific density of 1.18 at 20 °C). Each fecal sample was examined in duplicate under a light microscope (Olympus CX 23, Olympus, Tokyo, Japan) at magnifications of 100× and 400×. All eggs found were photographed and identified according to their morphological characteristics [22].

### 2.3. Risk Factors Assessment

In this study, we investigated the influence of various individual factors and environmental variables. The analysis of individual variables encompassed the following parameters: sex (male or female), age (<1 year, 1–5 years, 5–10 years, >10 years), size (<25 kg and >25 kg) and coat length (short, medium and long hair). The analysis of environmental variables considered the following parameters: attitude (pet, hunting, guard), diet (commercial, mixed, combined), habitat (indoor, outdoor, indoor/outdoor) and contact with other animals (yes or no).

The category of “pet dogs” includes animals kept in households for companionship, as well as those under the owner’s care with restricted movement. “Hunting dogs” are animals owned and maintained by hunters, assisting in locating, chasing, and recovering prey during hunting activities. The “guard dogs” category comprises dogs protecting property in yards, with controlled or partially controlled movement [12].

Commercial diets included branded foods designed to meet the nutritional requirements of pets for each stage of life or lifestyle. A mixed diet implied the consumption of different foods (such as raw meat, offal and bread) and access to paratenic or intermediate hosts. A combined diet included both, commercial and mixed diet.

### 2.4. Statistical Analyses

Results were analyzed using Graph Pad Prism software, version 7 (GraphPad, San Diego, CA, USA). Factors (individual and environmental variables) associated with parasitism were analyzed using the Chi-Square (X^2^) test. The odds ratio (OR) was calculated to verify the level of risk associated with variables that correlated with parasitism. To calculate the odds ratio, the following formula was used: *p* ± Z (*p* × (1 − *p*)/*n*) × 0.5, where *p* is prevalence, Z is the multiplier from the normal distribution at a 95% confidence interval (1.96) and *n* is the number of examined samples. In all analyses, the confidence level was 95%, and statistical analyses were considered significant if *p* < 0.05, *p* < 0.01 and *p* < 0.001.

## 3. Results

### 3.1. Prevalence of Gastrointestinal Parasites

**Figure 2:**
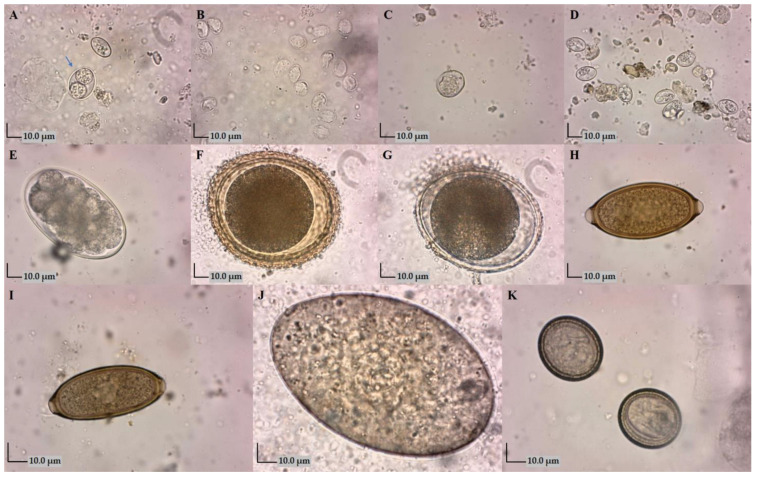
Parasitic elements detected in fecal samples, zinc sulphate flotation (×400): (**A**)—*Cystoisospora* spp. oocyst (blue arrow); (**B**)—*Giardia intestinalis* cysts; (**C**)—*Neospora caninum*/*Hammondia* spp. oocyst; (**D**)—*Sarcocystis* spp. sporocysts; (**E**)—Ancylostomatidae egg; (**F**)—*Toxocara canis* egg; (**G**)—*Toxascaris leonina* egg; (**H**)—*Trichuris vulpis* egg; (**I**)—*Capillaria* spp. egg; (**J**)—*Alaria alata* egg; (**K**)—Taeniidae eggs.

Through copromicroscopic investigation of fecal samples, endoparasites were found with a total prevalence of 62.6% (239/382). The prevalence of infections caused by protozoa was 12.3% (47/382), helminths 37.7% (144/382) and co-infection with protozoa and helminths was 12.6% (48/382). Eleven different species, genera or families of intestinal parasites were detected (Figure 2). The most prevalent protozoa was *Giardia intestinalis* (11.8%, 45/382). The presence of oocysts of *Cystoisospora* spp. (9.2%, 35/382), *Sarcocystis* spp. (4.5%, 17/382) and *Neospora caninum*/*Hammondia* spp. (3.7%, 14/382) were also detected. Of the nematodes, the most prevalent were Ancylostomatidae (38.0%, 145/382) and *Trichuris vulpis* (21.5%, 82/382), followed by *Toxocara canis* (11.5%, 44/382), *Capillaria* spp. (10.5%, 40/382) and *Toxascaris leonina* (4.2%, 16/382). Low prevalence of the trematode *Alaria alata* (1.6%, 6/382) and cestodes from the family Taeniidae (1.3%, 5/382) were also diagnosed. The most prevalent were monoinfections of dogs (29.8%, 114/382), followed by infections with two (18.1%, 69/382), three (9.2%, 35/382), four (2.1%, 8/382), five (2.62%, 10/382) and six (0.3%, 1/382) types of parasites (Table 1).

### 3.2. Individual Fisk Factors

The prevalence of endoparasitic infections was higher in male dogs (64.2%, 122/190) than in female dogs (60.9%, 117/192). Regarding the age of the dogs, a significantly higher prevalence of endoparasites (*p* < 0.001) was recorded in dogs younger than 1 year (83.3%, 55/66) compared to dogs aged 1–5 years (64.4%, 239/216), aged 5–10 years (46.2%, 36/78) and those older than 10 years (40.9%, 9/22) (Table 2). The prevalence of *G. intestinalis* (36.36%, 24/66), *T. canis* (27.27%, 18/64) and *T. leonina* (10.61%, 7/66) was significantly higher (*p* < 0.05; *p* < 0.001) in dogs <1 year, while a significantly higher (*p* < 0.05) prevalence of Ancylostomatidae was found in dogs <1 year and 1–5 years (Table 3). Endoparasitic infections were significantly higher (*p* < 0.05) in dogs weighing less than 25 kg (67.2%, 154/229) compared to those weighing over 25 kg (55.6%, 85/153) (Table 2). A significantly higher (*p* < 0.05) prevalence of *T. canis* (14.41%, 33/229), *T. leonina* (6.11%, 14/229) and Ancylostomatidae (41.92%, 96/229) was recorded in dogs weighing less than 25 kg (Table 4). Prevalence of endoparasites was significantly higher (*p* < 0.001) in short-haired dogs (67.2%, 160/238) compared to medium-haired (60.7%, 54/89) and long-haired dogs (45.5%, 25/55) (Table 2). The prevalence of Ancylostomatidae (42.86%, 102/238) and *Capillaria* spp. (13.87%, 33/238) was significantly higher (*p* < 0.05) in short-haired dogs (Table 4).

### 3.3. Environmental Risk Factors

Gastrointestinal parasites were more prevalent among dogs that were living with other animals (63.2%, 172/272) compared to those that were not (60.9%, 67/110) (Table 2). A significantly higher prevalence (*p* < 0.05, *p* < 0.01, *p* < 0.001) of *Cystoisospora* spp. (11.76%, 32/272), *Sarcocystis* spp. (5.88%, 16/272), *G. intestinalis* (15.07%, 41/272) and *T. canis* (14.34%, 39/272) was found among dogs that were living with other animals. On the contrary, a significantly higher prevalence (*p* < 0.05, *p* < 0.01) of Ancylostomatidae (50.0%, 55/110) and *Capillaria* spp. (15.45%, 17/110) was observed among dogs without contact with other animals (Table 5). Considering attitude, the prevalence of endoparasites was significantly higher (*p* < 0.001) among hunting dogs (81.6%, 120/147) compared to guard dogs (59.8%, 55/92) and pets (44.8%, 64/143) (Table 2). Among hunting dogs, a significantly higher prevalence (*p* < 0.05, *p* < 0.01, *p* < 0.001) was found for *Sarcocystis* spp. (9.52%, 14/147), *T. canis* (17.01%, 25/147), *T. leonina* (8.16%, 12/147), Ancylostomatidae (61.22%, 90/147), *T. vulpis* (34.01%, 50/147) and *Capillaria* spp. (17.69%, 26/147) (Table 5).

A significantly higher (*p* < 0.001) prevalence of parasites was recorded in the category of indoor/outdoor dogs (72.0%, 144/200) compared to outdoor (67.7%, 86/127) and indoor (16.4%, 9/55) dogs (Table 2). A significantly higher prevalence (*p* < 0.05; *p* < 0.01; *p* < 0.001) of *Cystoisospora* spp. (15.75%, 20/127), *Sarcocystis* spp. (8.66%, 11/127), *N. caninum*/*Hammondia* spp. (7.09%, 9/127) and *Alaria alata* (3.94%, 5/127) was found in the outdoor dog category. On the contrary, a significantly higher prevalence (*p* < 0.05; *p* < 0.01; *p* < 0.001) of *G. intestinalis* (17.5%, 35/200), Ancylostomatidae (45.5%, 91/200), *T. vulpis* (26.0%, 52/200) and *Capillaria* spp. (13.5%, 27/200) was found in the category of indoor/outdoor dogs (Table 6). In the category of dogs consuming mixed food (72.5%, 145/200), the prevalence of endoparasites was significantly higher (*p* < 0.001) compared to dogs consuming combined food (56.3%, 54/96) or commercial food (46.5%, 40/86) (Table 2). The prevalence of *Cystoisospora* spp. (13.5%, 27/200), *Sarcocystis* spp. (7.0%, 14/200), Ancylostomatidae (51.0%, 102/200) and *T. vulpis* (29.0%, 58/200) was significantly higher (*p* < 0.05; *p* < 0.01; *p* < 0.001) in dogs fed with a mixed diet, while the prevalence of *G. intestinalis* was significantly higher (*p* < 0.05) in dogs fed with a combined diet (Table 6).

## 4. Discussion

In our research, the total prevalence of gastrointestinal parasites in owned dogs was 62.6%. This finding is in accordance with previous research on dogs in public shelters in Serbia [3], which reported a total GI parasite prevalence of 58.3%. The results of numerous studies conducted in European countries reveal the different prevalence of endoparasites in dogs. In Greece [23,24], the prevalence ranged from 26% to 65%, in Slovakia from 27.1% to 45.7% [12,25], in Spain 53.6% [26], in Portugal from 41.0 to 81.19% [27,28,29] and in Germany 41.2% [30]. From the total number of examined fecal samples, the most frequent findings were monoinfections (29.8%), followed by infections with two (18.1%), three (9.2%), four (2.1%), five (2.62%), and six (0.3%) endoparasites. Similar to our findings, other studies have reported monoinfections as the most prevalent, while polyparasitism was also confirmed [8,12,27,31,32,33,34]. The prevalence of infections caused by protozoa in dogs in this research was 12.3%, helminths 37.7%, and co-infections with both protozoa and helminths 12.6%. A study from Spain found a higher prevalence of helminths (63.6%) in hunting dogs compared to intestinal protozoa (20.4%). In contrast, dogs from shelters had a higher prevalence of intestinal protozoa (67.9%) than helminths (9.8%) [35]. The heterogeneity of the available results depends on the origin of the samples (farm dogs, hunting dogs, owned dogs, shelter dogs, stray dogs) and the socio-economic status of the countries where the research was carried out [20].

### 4.1. Protozoa

Among the protozoa, *Giardia intestinalis* was the most prevalent (11.8%). It is widely reported in both domestic and wild animals, which can serve as hosts and reservoirs of zoonotic Assemblages [36,37,38,39]. This parasite is among the most common in humans, with an estimated 200 million people infected [40]. The prevalence of giardiosis in humans in developed countries ranges between 2 and 7%, and in developing countries 20 and 30% [41]. In this research, *G. intestinalis* was the most prevalent protozoa in dogs younger than one year (36.36%). Our results are in accordance with the results in the study by Murnik et al. [30], where the prevalence of *G. intestinalis* was 29%. An increased risk of giardiosis in dogs younger than one year has been confirmed in studies by other authors [42,43,44,45]. A higher prevalence was detected among the category of guard dogs and pets, as well as those who lived indoors/outdoors. Additionally, dogs that were fed commercial or combined diets and were in contact with other animals had a higher prevalence. Given the various ways *G. intestinalis* can spread through contaminated food and water [46,47,48], it is clear that these specific groups of dogs can serve as a source of environmental contamination, posing an indirect threat to individuals, particularly farmers, veterinarians, and animal handlers [41].

Oocysts of *Cystoisospora* spp. were identified in 9.2% of the samples examined. Oocysts were found most frequently in dogs younger than one year. The higher prevalence of *Cystoisospora* spp. found in younger dogs was confirmed in our previous study [3]. These results are also in accordance with Papazahariadou et al. [23], who reported a significantly higher number of coccidiosis cases in young dogs compared to adults. In addition, a higher prevalence of *Cystoisospora* spp. was found in dogs that live outside, have contact with other animals and consume mixed diets. This finding may be associated with the contaminated environment and the presence of this protozoa in the soil [15].

Among protozoa, a lower prevalence of *Sarcocystis* spp. (4.5%) and *Neospora caninum*/*Hammondia* spp. (3.7%) was found. The highest prevalence of *Sarcocystis* spp. was diagnosed in the category of hunting dogs (9.52%), which is not in accordance with results from Germany, where a high prevalence of sarcocystosis (63.3%) was found in hunting dogs in areas inhabited by wolves [49]. In that research, prevalence was determined using molecular methods, which is a more sensitive method than microscopical examination. Such differences could be explained by the assumption that the investigated hunting dogs originated from areas where wolves live. Compared to pet dogs in Germany, where the prevalence of sarcocystosis ranged from 2 to 9% [50], we found a lower prevalence in both pet dogs (1.40%) and guard dogs (1.09%) in our study. A higher prevalence was found in dogs that live outdoors, have contact with other animals and consume mixed diets. Such dogs are allowed to feed on the meat of herbivores, which are intermediate hosts for these protozoa, thus maintaining the circulation of this parasite [49,51].

Oocysts of *N. caninum*/*Hammondia* spp. were the most frequent in the category of dogs living outside (7.09%). Dogs fed a mixed diet had the highest number of positive samples (5.5%). This is likely because these dogs have the opportunity to consume infected tissues (raw or undercooked meat, fetal membranes) or intermediate hosts containing tissue cysts [52]. Given that *N. caninum* can cause abortions in cattle and cause economic loses in livestock, this category of dogs may pose a risk to cattle health. This risk is supported by the findings of Klun et al. [53], who reported a seroprevalence of this coccidia of 7.2% in cattle in Serbia.

### 4.2. Nematoda

The most common GI parasites identified in our study were hookworms from the Ancylostomatidae family (38.0%). This finding is in accordance with results from Bulgaria, where these parasites were most prevalent in owned and stray dogs, dogs that live outside and harbor dogs [54,55,56]. In our previous study on dogs from public shelters [3], the prevalence of Ancylostomatidae was 15.4%. A significantly higher prevalence of parasites was found in the category of dogs younger than one year and aged from 1 to 5 years, short-haired dogs and dogs lighter than 25 kg. Also, a higher prevalence of these nematodes was found in hunting dogs, dogs fed mixed diets and those living indoors/outdoors. Our finding aligns with Letra Mateus et al. [27], who reported a high prevalence of Ancylostomatidae in hunting dogs. This could be due to the dogs being kept together in groups and creating a favorable environment for parasite transmission. Also, factors such as hunting prey and consuming a wider variety of food sources might contribute to a higher risk of infection [9]. Rubel et al. [57] reported that the prevalence of hookworm is higher in regions with lower socio-economic status. On the contrary, in a study in Germany, in dogs younger than one year, the prevalence of these parasites was 0.9% [30]. Nematodes from the Ancylostomatidae family pose a risk to human health, given that their infectious stage can cause cutaneous larva migrans, and in the case of *Ancylostoma caninum*, eosinophilic enteritis or neuroretinitis [58,59,60].

*Trichuris vulpis* was the second most common parasite and was diagnosed in 21.5% of examined dogs. In research conducted in Bulgaria, this nematode was found in 15.1% of owned dogs kept outdoors [56] and 20% of dogs from shelters [54]. Additionally, it was found in 13.6% of dogs from shelters in Italy [32] and in 20% of dogs in Romania [61]. A lower prevalence was observed in 9.5% of owned dogs in Albania [62], 9.6% of hunting and herding dogs in Greece [23] and 4.8% of domestic dogs, along with 13.6% of shelter dogs in Italy [32]. In research conducted in Spain [35] and Portugal [27], trichuriosis was the most prevalent in hunting dogs, similar to our study (34.01%). A higher prevalence of *T. vulpis* was found in the category of dogs using mixed and combined food. The eggs of these parasites can remain viable for years, contaminating the environment, food and water, thereby posing a risk for infections in dogs [63].

*Toxocara canis* was found in 11.5% of the examined samples, with the highest prevalence in the population of hunting dogs (17.01%) and in dogs younger than one year (27.27%). The obtained results are consistent with findings from Europe, where *T. canis* prevalence ranged from 17.72% in Spain [64] to 11.9% to 16.5% in Slovakia [13,65], 12.8% in Greece [23], 5.1% to 11.28% in Portugal [27,28], 8% in Albania [62] and 6.4% in Bulgaria [56]. Comparing this with previous research conducted in Serbia, a higher prevalence of toxocarosis in pet dogs was observed at 16.6% [2], while in owned dogs that visit public parks it ranged from 36.6% to 38% [4], and in dogs from shelters it was 33.5% [3]. The larvae of this ascarid may pose a risk to humans, as upon infection they migrate into internal organs, potentially leading to visceral and ocular larva migrans [66]. In this regard, Deutz et al. [67] confirmed a high seroprevalence of *T. canis* among farmers, slaughterhouse staff, veterinarians and hunters.

Eggs of the trichurid type, exhibiting morphological characteristics specific to species from the genus *Capillaria*, were diagnosed in 10.5% of the fecal samples, with the assumption that they belong to a species of *C. aerophila*. The prevalence of *C. aerophila* in dogs across Europe and the Balkan countries varies, ranging from 0.4% to 0.5% in Italy [32], 0.65% in Romania [61], 2.8% in Albania [62] and from 2% to 11% in Bulgaria [55]. The prevalence of respiratory capillariosis in dogs cannot be determined with certainty, as the excreted eggs may not exclusively originate from adult parasites inhabiting the trachea. They could also appear in feces due to coprophagia or ingestion of food previously contaminated with eggs of *Capillaria* spp. from the feces of other dogs or animals [62]. A Higher prevalence of *Capillaria* spp. was found in the category of hunting dogs, those who live outside and those in contact with other animals. These results are not surprising, since a higher prevalence of *C. aerophila* (38%) was found in red foxes in Serbia [68].

The prevalence of *Toxascaris leonina* species in owned dogs was 4.2%. The highest number of positive findings was observed in hunting dogs (17.01%) and in the category of dogs younger than one year (10.61%). Ilić et al. [3] reported a prevalence of toxascarosis of 3.4% in dogs from public shelters in Serbia, while authors from Slovakia found this ascarid in 1.6% of various categories of dogs [12].

### 4.3. Trematoda and Cestoda

Among the other parasites, a lower prevalence of the trematode *Alaria alata* (1.6%) was diagnosed in this study. Besides wild carnivores, which are definitive hosts and contribute to the spread of *A. alata* [69], this parasite was confirmed in our study among hunting and guard dogs, as well as in dogs that live outdoors. The presence of *A. alata* was also found in the category of dogs that were fed with a mixed and combined diet. However, one positive sample was recorded in a dog fed commercial food, suggesting that the infection occurred after the consumption of intermediate hosts while the dog was outside.

Cestodes from the family Taeniidae were confirmed in five dogs (1.3%), which is slightly lower than the prevalence found in different categories of dogs (4%) in Slovakia [12] and in Germany (up to 12.2%) [30,49]. The positive samples were mostly obtained from hunting dogs that frequently stay in the wild during hunting, which is why they are at a higher risk of consuming intermediate hosts [27,70]. Although the eggs of species from the family Taeniidae cannot be differentiated by light microscopy, in veterinary medicine, as a precaution, any eggs of the taeniid type found are considered as the presence of eggs of the species *Echinococcus granulosus*. The presence of *E. granulosus* in the feces of owned dogs is particularly important for public health.

## 5. Conclusions

In the research, the total prevalence of endoparasites was 62.6%. Of particular importance for public health is the discovery of the largest number of gastrointestinal parasites found in categories of dogs younger than one year, hunting dogs, dogs kept indoors/outdoors and those fed with mixed food. Considering the finding of zoonotic endoparasites and the presence of species with zoonotic potential, the obtained results are particularly important for owners and veterinarians in clinical practice. These findings can aid in the adequate selection of antiparasitics, planning of deworming regimens and implementation of programs for the prevention of parasitic infections in dogs.

## Figures and Tables

**Table 1:** Prevalence of intestinal parasites.

Endoparasites	*n* = 382		
	Positive Samples	%	95% CI
*Cystoisospora* spp.	35	9.2	6.30–12.10
*Sarcocystis* spp.	17	4.5	2.42–6.58
*Neospora caninum*/*Hammondia* spp.	14	3.7	1.81–5.69
*Giardia intestinalis*	45	11.8	8.56–15.04
*Toxocara canis*	44	11.5	8.30–14.70
*Toxascaris leonina*	16	4.2	2.10–6.20
Ancylostomatidae	145	38.0	33.13–42.87
*Trichuris vulpis*	82	21.5	17.38–25.62
*Capillaria* spp.	40	10.5	7.43–13.57
*Alaria alata*	6	1.6	0.34–2.86
Taeniidae	5	1.3	0.16–2.44
**Occurrence of Infections**	***n* = 382**		
	**Positive Samples**	**%**	**95% CI**
Protozoa	47	12.3	9.01–15.59
Helminths	144	37.7	32.84–42.56
Protozoa + Helminths	48	12.6	9.06–16.14
**Occurrence of Mixed Infections**	***n* = 382**		
	**Positive Samples**	**%**	**95% CI**
With one parasite	114	29.84	25.25–34.43
With two parasites	69	18.06	14.20–21.92
With three parasites	35	9.16	6.27–12.05
With four parasites	8	2.09	0.66–3.52
With five parasites	10	2.62	1.02–4.22
With six parasites	1	0.26	0–0.77

*n*—number of examined samples, CI—Confidence interval.

**Table 2:** Individual and environmental risk factors.

		*n*	*N*	%	χ^2^	*p*	Odds Ratio
Individual risk factors	Sex						
	Male	190	122	64.2	0.44	0.51	1.15
	Female	192	117	60.9			
	Size						
	<25 kg	229	154	67.2	5.36	*	1.64
	>25 kg	153	85	55.6			
	Age						
	<1 year	66	55	83.3	25.85	***	
	1–5 year	216	139	64.4			2.77
	5–10 year	78	36	46.2			5.83
	>10 year	22	9	40.9			7.22
	Coat length						
	Short	238	160	67.2	9.22		
	Medium	89	54	60.7		***	1.33
	Long	55	25	45.5			2.96
Environmental risk factors	Living with other animals						
	Yes	272	172	63.2	0.45	0.50	0.85
	No	110	67	60.9			
	Habitat						
	Indoor	55	9	16.4	59.17	***	
	Outdoor	127	86	67.7			0.09
	Indoor/Outdoor	200	144	72.0			0.07
	Diet						
	Commercial	86	40	46.5	19.53	***	
	Mixed food	200	145	72.5			0.33
	Combined	96	54	56.3			0.68
	Attitude						
	Pet	143	64	44.8	42.49	***	
	Guard dog	92	55	59.8			0.54
	Hunting dog	147	120	81.6			0.18

*n*—number of examined samples; *N*—number of positive samples; * *p* < 0.05; *** *p*< 0.001.

**Table 3:** Influence of individual risk factors (sex and age) on prevalence of intestinal parasites.

	Sex				χ^2^	*p*	Age								χ^2^	*p*
	Male		Female				<1 Year			1–5 Year		5–10 Year		>10 Year		
*n*	229		153				66			216		78		22		
End	*N*	% (95% CI)	*N*	% (95% CI)			*N*	% (95% CI)	*N*	% (95% CI)	*N*	% (95% CI)	*N*	% (95% CI)		
Cys	19	10.00 (5.73–14.27)	16	8.33 (4.42–12.24)	0.32	0.57	11	16.67 (10.99–22.35)	19	8.80 (5.02–12.58)	4	5.13 (0.23–9.13)	1	4.55 (0–13.26)	6.59	0.09
Sar	8	4.21 (1.36–7.06)	9	4.69 (1.70–7.68)	0.05	0.82	2	3.03 (0–7.17)	10	4.63 (1.83–7.43)	5	6.41 (1.06–11.76)	0	0.00	2.06	0.56
Neo	7	3.68 (1–6.36)	7	3.65 (1–6.31)	0	1	2	3.03 (0–7.17)	7	3.24 (0.88–5.60)	3	3.85 (0–8.05)	2	9.09 (0–21.10)	2.03	2.03
Gia	22	11.58 (7.03–16.13)	23	11.98 (7.39–16.57)	0.02	0.91	24	36.36 (24.75–47.97)	18	8.33 (5.11–12.02)	2	2.56 (0–6.07)	1	4.55 (0–13.26)	48.33	***
Tox	21	11.05 (6.59–15.51)	23	11.98 (7.39–16.57)	0.08	0.78	18	27.27 (16.53–38.01)	21	9.72 (5.77–13.67)	4	5.13 (0.23–10.03)	1	4.55 (0–13.26)	20.93	***
Tas	5	2.63 (0.35–4.49)	11	5.73 (2.44–9.02)	2.28	0.13	7	10.61 (3.18–18.04)	7	3.24 (0.88–5.60)	2	2.56 (0–6.07)	0	0.00	8.73	*
Anc	75	39.47 (32.52–46.42)	70	36.46 (29.65–43.27)	0.37	0.54	27	40.91 (29.05–52.77)	93	43.06 (36.46–49.66)	20	25.64 (15.95–35.33)	5	22.73 (5.22–40.24)	9.82	*
Tri	41	21.58 (15.73–27.43)	41	21.35 (8.81–33.89)	0	1	13	19.70 (10.10–29.30)	49	22.69 (17.10–28.28)	16	20.51 (11.55–29.47)	4	18.18 (2.07–34.29)	0.50	0.92
Cap	22	11.58 (7.03–16.13)	18	9.38 (5.26–13.50)	0.50	0.48	8	12.12 (4.25–19.99)	28	12.96 (8.48–17.44)	4	5.13 (0.23–10.03)	0	0.00	6.57	0.09
Ala	3	1.58 (0–3.35)	3	1.56 (0–3.31)	0	1	1	1.52 (0–4.47)	2	0.93 (0–2.21)	2	2.56 (0–6.07)	1	4.55 (0–13.26)	2.34	0.51
Tae	3	1.58 (0–3.35)	2	1.04 (0–2.48)	0.21	0.64	0	0.00	5	2.31 (0.31–4.31)	0	0.00	0	0.00	3.89	0.27

*n*—number of examined samples; *N*—number of positive samples; CI—Confidence interval; * *p* < 0.05; *** *p*< 0.001; Cys—*Cystoisospora* spp.; Sar—*Sarcocystis* spp.; Neo—*Neospora caninum*/*Hammondia* spp.; Gia—*Giardia intestinalis*; Tox—*Toxocara canis*; Tas—*Toxascaris leonina*; Anc—Ancylostomatidae; Tri—*Trichuris vulpis*; Cap—*Capillaria* spp.; Ala—*Alaria alata*; Tae—Taeniidae.

**Table 4:** Influence of individual risk factors (size and coat length) on prevalence of intestinal parasites.

	Size				χ^2^	*p*	Coat Length						χ^2^	*p*
	<25 kg		>25 kg				Short		Medium		Long			
*n*	229		153				238		89		55			
End	*N*	% (95% CI)	*N*	% (95% CI)			*N*	% (95% CI)	*N*	% (95% CI)	*N*	% (95% CI)		
Cys	23	10.04 (6.15–13.93)	12	7.84 (3.54–12.14)	0.53	0.46	17	7.14 (3.77–10.51)	13	14.61 (7.27–21.94)	5	9.09 (1.49–16.69)	4.34	0.11
Sar	11	4.80 (2.03–7.57)	6	3.92 (0.84–7.00)	0.17	0.68	12	5.04 (2.26–7.82)	5	5.62 (0.83–10.40)	0	0.00	3.04	0.22
Neo	9	3.93 (1.41–6.45)	5	3.27 (0.45–6.09)	0.11	0.74	9	3.78 (1.36–6.20)	3	3.37 (0–7.12)	2	3.64 (0–8.59)	0.03	0.98
Gia	30	13.10 (8.70–17.50)	15	9.80 (5.09–14.51)	0.96	0.33	30	12.61 (8.39–16.83)	11	12.36 (5.52–19.22)	4	7.27 (0.41–14.13)	1.26	0.53
Tox	33	14.41 (9.86–18.96)	11	7.19 (3.10–11.28)	4.69	*	29	12.18 (8.02–16.34)	11	12.36 (5.52–19.22)	4	7.27 (0.41–14.13)	1.14	0.57
Tas	14	6.11 (3.01–9.21)	2	1.31 (0–3.11)	5.28	*	9	3.78 (1.36–6.20)	6	6.74 (1.53–11.95)	1	1.82 (0–5.35)	2.31	0.31
Anc	96	41.92 (35.53–48.31)	49	32.03 (24.64–39.42)	3.81	*	102	42.86 (36.57–49.15)	28	31.46 (21.81–41.61)	15	27.27 (15.50–39.04)	6.69	*
Tri	53	23.14 (17.68–28.60)	29	18.95 (12.74–15.16)	0.96	0.33	55	23.11 (17.75–28.47)	19	21.35 (12.84–29.86)	8	14.55 (5.23–23.87)	1.95	0.38
Cap	28	12.23 (7.99–16.47)	12	7.84 (3.54–12.14)	1.88	0.17	33	13.87 (9.48–18.26)	5	5.62 (0.83–10.40)	2	3.64 (0–8.59)	7.90	*
Ala	3	1.31 (0–2.78)	3	1.96 (0–4.16)	0	1	5	2.10 (0.27–3.91)	1	1.12 (0–3.31)	0	0.00	1.43	0.49
Tae	3	1.31 (0–2.78)	2	1.31 (0–3.11)	0	1	4	1.68 (0.05–3.31)	0	0.00	1	1.82 (0–5.35)	1.55	0.46

*n*—number of examined samples; *N*—number of positive samples; CI—Confidence interval; * *p* < 0.05; Cys—*Cystoisospora* spp.; Sar—*Sarcocystis* spp.; Neo—*Neospora caninum*/*Hammondia* spp.; Gia—*Giardia intestinalis*; Tox—*Toxocara canis*; Tas—*Toxascaris leonina*; Anc—Ancylostomatidae; Tri—*Trichuris vulpis*; Cap—*Capillaria* spp.; Ala—*Alaria alata*; Tae—Taeniidae.

**Table 5:** Influence of environmental risk factors (living with other animals and attitude) on prevalence of intestinal parasites.

	Living with Other Animals				χ^2^	*p*	Attitude						χ^2^	*p*
	Yes		No				Pet		Guard Dog		Hunting Dog			
*n*	272		110				143		92		147			
End	*N*	% (95% CI)	*N*	% (95% CI)			*N*	% (95% CI)	*N*	% (95% CI)	*N*	% (95% CI)		
Cys	32	11.76 (7.93–15.59)	3	2.73 (0–5.77)	7.69	**	12	8.39 (3.85–12.93)	7	7.61 (2.19–13.03)	16	10.88 (5.85–15.91)	12	0.64
Sar	16	5.88 (3.08–8.68)	1	0.91 (0–2.68)	4.56	*	2	1.40 (0–3.33)	1	1.09 (0–2.96)	14	9.52 (4.78–14.26)	2	***
Neo	11	4.04 (1.70–6.38)	3	2.73 (0–5.77)	0.39	0.53	6	4.20 (0.91–7.49)	4	4.35 (0.18–8.52)	4	2.72 (0.10–5.34)	6	0.74
Gia	41	15.07 (10.82–19.32)	4	3.64 (0.14–7.14)	9.86	***	18	12.59 (7.15–18.03)	13	14.13 (7.01–21.25)	14	9.52 (4.78–14.26)	18	0.52
Tox	39	14.34 (10.17–18.51)	5	4.55 (0.66–8.44)	7.37	**	10	6.99 (2.81–11.17)	9	9.78 (3.71–15.85)	25	17.01 (10.94–23.08)	10	*
Tas	12	4.41 (1.97–6.85)	4	3.64 (0.14–7.14)	0.12	0.73	1	0.70 (0–2.56)	3	3.26 (0–6.89)	12	8.16 (3.73–12.58)	1	**
Anc	90	33.09 (27.50–39.68)	55	50.00 (40.66–59.34)	9.51	**	23	16.08 (10.06–22.10)	32	34.78 (25.05–44.51)	90	61.22 (53.34–69.10)	23	***
Tri	54	19.85 (15.11–24.59)	28	25.45 (17.31–33.59)	1.46	0.23	14	9.79 (4.92–14.66)	18	19.57 (11.46–27.68)	50	34.01 (26.35–41.67)	14	***
Cap	23	8.46 (5.15–11.77)	17	15.45 (8.70–22.20)	4.09	*	7	4.90 (1.36–8.44)	7	7.61 (2.19–13.03)	26	17.69 (11.52–23.86)	7	***
Ala	6	2.21 (0.46–3.96)	0	0.00	2.47	0.12	1	0.70 (0–2.56)	2	2.17 (0–5.15)	3	2.04 (0–4.33)	1	0.57
Tae	3	1.10 (0–2.34)	2	1.82 (0–4.32)	0.31	0.58	1	0.70 (0–2.56)	0	0.00	4	2.72 (0.10–5.34)	1	0.14

*n*—number of examined samples; *N*—number of positive samples; CI—Confidence interval; * *p* < 0.05; ** *p* < 0.01; *** *p* < 0.001; Cys—*Cystoisospora* spp.; Sar—*Sarcocystis* spp.; Neo—*Neospora caninum*/*Hammondia* spp.; Gia—*Giardia intestinalis*; Tox—*Toxocara canis*; Tas—*Toxascaris leonina*; Anc—Ancylostomatidae; Tri—*Trichuris vulpis*; Cap—*Capillaria* spp.; Ala—*Alaria alata*; Tae—Taeniidae.

**Table 6:** Influence of environmental risk factors (habitat and diet) on prevalence of intestinal parasites.

	Habitat						χ^2^	*p*	Diet						χ^2^	*p*
	Indoor		Outdoor		Indoor/Outdoor				Commercial		Mixed Food		Combined			
*n*	55		127		200				86		382		96			
End	*N*	% (95% CI)	*N*	% (95% CI)	*N*	% (95% CI)			*N*	% (95% CI)	*N*	% (95% CI)	*N*	% (95% CI)		
Cys	1	1.82 (0–5.33)	20	15.75 (9.41–22.08)	14	7.00 (3.46–10.54)	11.31	**	3	3.49 (0–7.37)	27	13.5 (8.76–18.24)	5	5.21 (0.76–9.66)	9.65	***
Sar	0	0.00	11	8.66 (3.77–13.55)	6	3.00 (0.64–5.36)	8.85	*	0	0.00	14	7.0 (3.46–10.54)	3	3.13 (0–6.61)	7.46	*
Neo	0	0.00	9	7.09 (2.62–11.55)	5	2.50 (0.34–4.66)	7.07	*	3	3.49 (0–7.37)	11	5.5 (2.34–8.60)	0	0.00	5.57	0.06
Gia	4	7.27 (0.41–14.63)	6	4.72 (1.03–8.41)	35	17.50 (12.23–22.77)	13.46	***	16	18.60 (10.38–16.82)	15	7.5 (3.85–11.15)	14	14.58 (7.52–21.64)	8.11	*
Tox	3	5.45 (0–11.45)	17	13.39 (7.47–19.31)	24	12.00 (7.50–16.50)	2.46	0.29	7	8.14 (2.37–14.92)	27	13.5 (8.76–18.24)	10	10.42 (5.54–15.30)	1.85	0.40
Tas	1	1.82 (0–5.33)	5	3.94 (0.56–7.32)	10	5.00 (1.98–8.02)	1.12	0.57	4	4.65 (0.20–9.10)	9	4.5 (1.63–6.57)	3	3.13 (0–6.61)	0.37	0.83
Anc	2	3.64 (0–8.59)	52	40.94 (32.39–49.49)	91	45.50 (38.55–52.40)	32.82	***	16	18.60 (10.38–16.82)	102	51.0 (44.07–57.93)	27	28.13 (19.14–37.13)	32.07	***
Tri	1	1.82 (0–5.33)	29	22.83 (15.53–30.13)	52	26.00 (19.92–32.08)	15.18	***	5	5.81 (0.87–10.75)	58	29.0 (22.71–35.23)	19	19.79 (11.82–27.76)	19.39	***
Cap	0	0.00	13	10.24 (4.97–15.51)	27	13.50 (8.76–18.24)	8.40	*	8	9.30 (3.16–15.44)	25	12.5 (7.92–17.08)	7	7.29 (2.09–12.49)	2.04	0.36
Ala	0	0.00	5	3.94 (0.56–7.32)	1	0.50 (0–1.48)	6.96	*	1	1.16 (0–3.42)	3	1.5 (0–3.18)	2	2.08 (0–4.93)	0.26	0.88
Tae	0	0.00	2	1.57 (0–3.73)	3	1.50 (0–3.18)	0.86	0.65	0	0.00	4	2.0 (0.06–3.94)	1	1.04 (0–3.07)	1.93	0.38

*n*—number of examined samples; *N*—number of positive samples; CI—Confidence interval; * *p* < 0.05; ** *p* < 0.01; *** *p* < 0.001; Cys—*Cystoisospora* spp.; Sar—*Sarcocystis* spp.; Neo—*Neospora caninum*/*Hammondia* spp.; Gia—*Giardia intestinalis*; Tox—*Toxocara canis*; Tas—*Toxascaris leonina*; Anc—Ancylostomatidae; Tri—*Trichuris vulpis*; Cap—*Capillaria* spp.; Ala—*Alaria alata*; Tae—Taeniidae.

## Data Availability

The data presented in this study are available on request from the corresponding author.
